# Lower body muscle strength, dynapenic obesity and risk of type 2 diabetes –longitudinal results on the chair-stand test from the Survey of Health, Ageing and Retirement in Europe (SHARE)

**DOI:** 10.1186/s12877-022-03647-7

**Published:** 2022-12-01

**Authors:** Bernd Kowall

**Affiliations:** Institute of Medical Informatics, Biometry and Epidemiology (IMIBE), University Hospital Essen, Hufelandstraße 55, 45147 Essen, Germany

**Keywords:** Chair-stand test, Diabetes mellitus, Dynapenia, Handgrip strength, Muscle strength, Sarcopenia

## Abstract

**Background:**

The chair-stand test is a measure of lower body muscle strength. In a longitudinal study with older adults, we investigated whether results of the five-repetition chair-stand test (CST-5) are associated with incident type 2 diabetes, and whether diabetes risk in obese persons is modified by dynapenia (age-related loss of muscle strength) in the lower limbs.

**Methods:**

We used data of the Survey of Health, Ageing and Retirement in Europe (SHARE), a panel study with eight waves carried out between 2004 and 2020 in 28 European countries and Israel mainly in persons aged 50 years or older. Forty-six thousand one hundred nineteen persons (mean age 63.5 years, 44.1% men) with CST-5 data and follow-up data for diabetes were included from wave 2 and waves 4 to 7. The mean follow-up time was 5.3 years (standard deviation 2.9 years). Relative risks with 95% confidence intervals (CI) were estimated from log-linear models with a Poisson working likelihood and robust standard errors.

**Results:**

In the crude model, increased risks of diabetes were found for persons who considered the CST-5 as not safe, or whose times for the test were in the highest or second highest quartiles (relative risks 2.18 (95% CI: 1.95–2.43), 1.71 (1.54–1.91), 1.44 (95% CI: 1.29–1.61), reference: lowest quartile). These associations were attenuated in the fully adjusted regression model (relative risks 1.32 (95% CI: 1.17–1.48), 1.23 (1.10–1.37), 1.19 (1.06–1.33)). Furthermore, in fully adjusted models, the risk of diabetes in obese persons did not depend on whether they had low muscle strength or not. In obese persons with times for 5 sits and stands > 15 seconds, the adjusted risk of diabetes was 2.56 (95% CI: 2.22–2.95) times higher than in non-obese persons with times ≤15 seconds. The corresponding relative risk in obese persons with times ≤15 seconds was 2.45 (2.25–2.67).

**Conclusions:**

Poor results in the CST-5 were associated with an increased risk of diabetes. Among obese persons, the risk of diabetes was not modified by results of the CST-5.

## Introduction

In 2021, 61.4 million people in the age of 20–79 years in Europe had diabetes which corresponds to a prevalence of 9.2% [1]. The prevalence of diabetes strongly increases with age: In German men, for example, the prevalence of diabetes in 2010 was 1.6% in 40-49 year old people, 5.7% in 50–59 year olds, 14.5% in 60-69 year olds, and 21.9% in 70–79 year olds (women: 1.3, 3.6, 10.0, 16.9%) [2]. Thus, the number of people with diabetes in ageing European countries is expected to increase further [1]. People with diabetes have a two-fold risk for vascular diseases compared to people without diabetes [3]. Moreover, persons with diabetes have a higher risk of premature death than persons without diabetes [4]. In Europe, in 20–79 year old adults, 8.5% of all-cause deaths are attributable to diabetes [5]. In view of the adverse health consequences of diabetes, it is important that type 2 diabetes can be prevented or delayed [6]. Therefore, it is meaningful to identify further risk factors for type 2 diabetes which may be useful to identify persons at risk of diabetes earlier, and to prevent or delay progression to diabetes.

The chair-stand test (CST) is a measure of lower body muscle strength which is mainly used in older persons [7]. Slow chair-standers have a higher risk of becoming disable to perform activities of daily life [8]. Additionally, the results of the 30 second CST (CST-30) predicts 10-year survival, and it is associated with depression, cognitive decline, and multimorbidity [9–11]. However, there is still a lack of longitudinal studies on poor lower body muscle strength and chronic diseases, and, in particular, associations between results of the CST and incident diabetes have not been reported so far.

In the English Longitudinal Study of Ageing, older persons with dynapenic obesity – this is a combination of low muscle strength and obesity – were reported to have an increased risk of type 2 diabetes, and, in fact, in obese study participants, those with low or intermediate handgrip strength had a higher diabetes risk than those with high handgrip strength [12]. An association between dynapenia and type 2 diabetes was also found in a cross-sectional study in overweight / obese Japanese men [13]. However, further longitudinal studies to confirm the effect of dynapenic obesity on incident diabetes are lacking.

Handgrip strength is another widely used measure of muscle strength. In a recent meta-analysis, handgrip strength was shown to be negatively associated with incident diabetes, albeit not in all studies [14]. Handgrip strength is often seen as a proxy of overall muscle strength, but there are also conflicting results suggesting that handgrip strength is rather a measure of upper body muscle strength [15–17]. Therefore, it is worthwhile to investigate how strongly results of the CST are correlated with results of the handgrip strength test (HGST). Moreover, it has been shown that handgrip strength leads to an improvement of discrimination (albeit small) when it is added to a diabetes prediction model which includes risk factors of type 2 diabetes which can be measured non-invasively [18]. So far, it is not known whether addition of CST to diabetes prediction models improves the predictive ability of the models.

In the present study, our aim was to investigate whether poor results in the five repetition CST (CST-5) and dynapenic obesity are associated with incident diabetes in a large, long lasting panel study. In particular, we aimed to investigate whether the risk of diabetes in obese persons is modified by results in the CST-5. Moreover, we examined whether results in the CST-5 improve discrimination of diabetes prediction models.

## Methods

### Study population and data analysis set

We used data of the Survey of Health, Ageing and Retirement in Europe (SHARE), a panel study with eight waves carried out between 2004 and 2020 in 28 European countries and Israel mainly in persons aged 50 years or older [19–22]. We used data from waves 2, 4, 5, 6 and 7 of SHARE which were collected in 2006/2007 (wave 2), 2011 (wave 4), 2013 (wave 5), 2015 (wave 6) and 2017 (wave 7) – these five waves include residents from 19 European countries and Israel. We did not take wave 3 into account because it has a focus on the life history of the participants and, thus, differs from the other waves. The CST-5 was performed only in waves 2 and 5. As we were interested in the results of the CST-5 as the exposure variable, wave 1 was not taken into account either. Participants were interviewed every 2 years. The mean follow-up time was 5.3 years (standard deviation (SD) 2.9 years). For participants with the CST-5 in wave 2, the mean follow-up was 8.3 years (SD 2.6 years), for those with the CST-5 in wave 5, it was 3.5 years (SD 1.0 years). The interviews covered a wide range of topics, including demographics, physical and mental health, cognitive function, health care, lifestyle, social support, housing, employment, pensions, household income, and financial transfers. The study rationale and design have been described elsewhere, and further information on SHARE is available online [19–22]. SHARE data are available free of charge after registration.

**Fig. 1:**
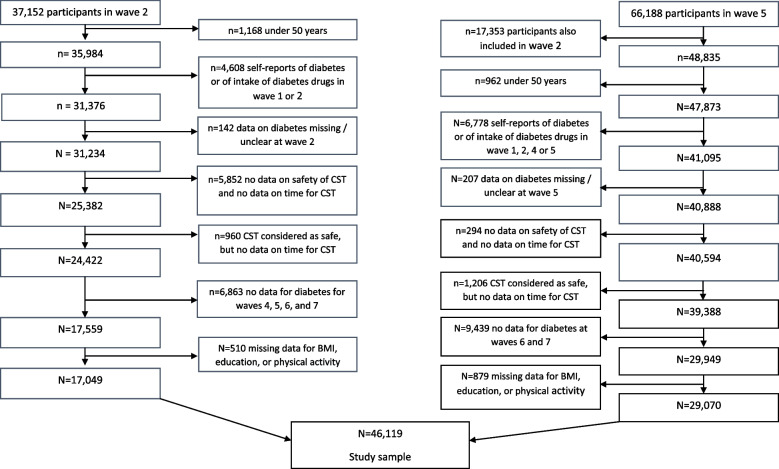
Flow chart for study participants with chair-stand test in wave 2 and wave 5

Seventeen thousand forty-nine participants of wave 2, and 29,070 of wave 5 were aged ≥50 years, neither had known diabetes nor took diabetes drugs at the time of the test, had all undergone the CST-5, were followed up in at least one more wave after performing the CST-5 and did not have missing values for education, BMI and physical activity (cf. flow chart in Fig. 1).

### Variables

#### CST-5

In the CST-5, the time to fully rise and sit down again five times in a row is recorded [23]. The equipment necessary to perform the test is very simple (a stop watch and a chair). The CST shows good test-retest reliability, and good construct validity [24]. In the CST-5, participants were asked to fold their arms across their chest and to sit so that their feet were on the floor, and then to stand up keeping their arms folded across their chest. This was demonstrated to the participants by the interviewer, and the participants were asked whether they felt safe to perform the test. If so, time for five sit and stands in a row was measured in seconds. From the results of the CST-5, a five-level categorical variable was built (test not safe; test safe and time in seconds in the lowest, second lowest, second highest, or highest sex-specific quartile). Longer times required to complete the CST-5 indicate a worse performance.

#### Handgrip strength

A handheld dynamometer (Smedley, S Dynamometer, TTM, Tokyo, 100 kg) was used to measure handgrip strength [25]. The test was performed in either a standing (preferred) or sitting position, with the elbow at a 90 degree angle, the upper arm tight against the trunk, and the wrist in a neutral position. The interviewers told the participants to squeeze the handle as hard as they could. Before the measurements, the participants were asked whether they were willing to have their handgrip measured. If so, they had a practice with one hand. Two alternate measurements were taken from the right and from the left hand. The maximum of the four measurements was used for further analysis. As result of the HGST, a five-level categorical variable was built in an analogous way as for CST-5.

#### Diabetes and comorbidities

To assess chronic diseases, participants were shown a card with 16 diseases (heart attack including myocardial infarction or coronary thrombosis or any other heart problem including congestive heart failure; high blood pressure or hypertension; high blood cholesterol; stroke or cerebral vascular disease; diabetes or high blood sugar; chronic lung disease such as chronic bronchitis or emphysema; asthma; arthritis; osteoporosis; cancer or malignant tumor; stomach or duodenal ulcer, peptic ulcer; Parkinson disease; cataracts; hip fracture or femoral fracture; other fractures; Alzheimer’s disease, dementia; benign tumor) and asked: “Has a doctor ever told you that you had/Do you currently have any of the conditions on this card? With this we mean that a doctor has told you that you have this condition, and that you are either currently being treated for or bothered by this condition. Please tell me the number or numbers of the conditions.” New-onset diabetes was assessed by two questions: one on whether a doctor had ever told the participants they had diabetes or high blood sugar, and one on the intake of diabetes drugs. We assume that incident diabetes at the age of 50 years or older is very likely to be type 2 diabetes.

#### Other covariables

The following socio-demographic variables were taken into account: age, sex, educational attainment and country of residence. Age was used as a continuous variable. International Standard Classification of Education codes (ISCED-97) were used which provide the following classification of educational level: pre-primary, primary, lower secondary, upper secondary, post-secondary non-tertiary, first stage of tertiary, second stage of tertiary, other [26]. We classified Austria, Germany, the Netherlands, France, Switzerland, Belgium, and Luxembourg as “Western Europe”; Sweden and Denmark as “Northern Europe”; Spain, Italy, Greece, Portugal, Croatia, and Israel as “Southern Europe”; Czech Republik, Poland, Hungary, Slovenia, and Estonia as “Eastern Europe”.

Body mass index (BMI) was calculated from self-reports of weight and height, and categorized according to WHO recommendations [27]. Furthermore, participants were asked how often they engaged in vigorous physical activity (more than once a week; once a week; one to three times a month; hardly ever or never). All variables were assessed at wave 2 or 5, depending on when the CST-5 was done for the first time.

### Statistical analyses

Relative risks with 95% CIs for the association between results of the CST-5 and incident diabetes were estimated from log-linear models with a Poisson working likelihood and robust standard errors. Three models were fitted: model 1: crude, model 2: adjusted for age (as a continuous variable) and sex, model 3: adjusted for age, sex, BMI (< 18.5, 18.5-24.9, 25-29.9, ≥ 30.0 kg/m^2^), vigorous physical activity, number of chronic diseases, education (ISCED1997), country group (as a categorical variable). An earlier study showed that age, sex, moderate or vigorous exercise, number of morbidity conditions, income, but not smoking, were associated with muscle strength [28]. Therefore, we did not include smoking in the adjustment set.

These regression models were fitted again to examine whether persons with obesity and dynapenia (age related loss of muscle strength) have a larger risk of type 2 diabetes than persons with dynapenia or obesity alone. Two analyses on this question were done:

In a first analysis, an exposure variable with eight categories was built from obesity (> 30 kg/m^2^_,_ ≤ 30 kg/m^2^) and time for CST-5 (considered as not safe; little (Q4), medium (Q2, Q3) or much (Q1) time needed). In a second analysis, the definition of the European Working Group on Sarcopenia in Older People (EWGSOP2) of low muscle strength was used (time for the CST-5 >  15 seconds) [29]. The exposure variable for this analysis has four categories: BMI > 30 kg/m^2^ and time >  15 s; BMI > 30 kg/m^2^ and time ≤ 15 s; BMI ≤ 30 kg/m^2^ and time >  15 s; BMI ≤ 30 kg/m^2^ and time ≤ 15 s.

To assess the ability of the CST-5 to predict diabetes, we used a logistic regression model with strong risk factors of diabetes which can be measured non-invasively (age, sex, BMI, vigorous physical activity, education, number of chronic diseases). We investigated how strongly the area under the receiver operating characteric curve (AROC) improved after adding the results of the CST-5.

To compare the five-level categorical variables for the CST-5 and the HGST, Cramer’s V was calculated. Pearson correlation coefficients were calculated between handgrip strength and time for five stands and sits in the CST-5.

For participants without incident diabetes, follow-up time was calculated as the time between the first wave (either wave 2 or wave 5) and the last wave in which the participant took part. For participants with incident diabetes, follow-up time was calculated as the time between the first wave and the onset time of diabetes. The midpoint between the wave where a participant reported diabetes diagnosis (or intake of diabetes drugs) for the first time and the previous wave in which the participant took part was used as time of diabetes onset.

All statistical analyses were performed using SAS Version 9.4 (SAS Institute, Cary, USA).

## Results

The study population included 46,119 participants (17,049 with baseline data form wave 2 and 29,070 participants with baseline data from wave 5) (cf. Fig. 1). The mean age of the participants at the time of their first CST-5 at wave 2 and wave 5, respectively, was 63.5 years, and 44.1% were men (Table 1). In men, 67.4% were overweight or obese and 69.7% reported at least one chronic disease; in women, 55.0% were overweight or obese, and 73.2% reported at least one chronic disease. In those who performed the HGST, the mean handgrip strength was higher in men than in women (44.9 kg versus 27.5 kg). The proportion of those not feeling safe to perform the CST-5 was slightly larger in women than in men (15.5% versus 12.7%), and among those able to perform the CST-5, men needed on average less time for five stands and sits than women (11.2 versus 12.2 seconds).

**Table 1:** Characteristics of study participants at the time of the chair-stand test: the Survey of Health, Ageing and Retirement (SHARE)

	Men	Women
N	20,357	25,762
Follow-up time	5.3 ± 2.9	5.3 ± 2.9
Age (years)	63.7 ± 8.8	63.3 ± 9.1
ISCED-97		
Pre-primary education	734 (3.6%)	959 (3.7%)
ISCED-97 code 1^a^	2954 (14.5%)	4589 (17.8%)
ISCED-97 code 2^b^	3182 (15.6%)	4570 (17.7%)
ISCED-97 code 3^c^	7100 (34.9%)	8542 (33.2%)
ISCED-97 code 4^d^	995 (4.9%)	1251 (4.9%)
ISCED-97 code 5^e^	5049 (24.8%)	5598 (21.7%)
ISCED-97 code 6^f^	267 (1.3%)	161 (0.6%)
Other	76 (0.4%)	92 (0.4%)
BMI (kg/m^2^)		
< 18.5	82 (0.4%)	436 (1.7%)
18.5 – 24.9	6539 (32.2%)	11,160 (43.3%)
25.0 – 29.9	10,064 (49.4%)	9307 (36.1%)
≥ 30	3672 (18.0%)	4859 (18.9%)
Number of chronic diseases		
0	6172 (30.3%)	6913 (26.8%)
1	6918 (34.0%)	8271 (32.1%)
2	4084 (20.1%)	5360 (20.8%)
3	1882 (9.2%)	3058 (11.9%)
4	811 (4.0%)	1274 (4.9%)
≥ 5	490 (2.4%)	886 (3.4%)
Vigorous sports or activities		
More than once a week	9000 (44.2%)	9094 (35.3%)
Once a week	2946 (14.5%)	4005 (15.6%)
1 to 3 times a month	1986 (9.8%)	2342 (9.1%)
Hardly ever / never	6425 (31.6%)	10,321 (40.1%)
Country		
Northern Europe	3092 (15.2%)	3559 (13.8%)
Western Europe	8333 (40.9%)	10,373 (40.3%)
Southern Europe	4731 (23.2%)	5571 (21.6%)
Eastern Europe	4201(20.6%)	6259 (24.3%)
Handgrip strength test		
Unable or refusing to perform the test	901 (4.4%)	1535 (6.0%)
Test performed	19,437 (95.6%)	24,206 (94.0%)
Mean handgrip strength (kg)	44.9 ± 9.7	27.5 ± 6.7
CST-5		
Did not feel safe to do the CST-5	2587 (12.7%)	4000 (15.5%)
Test performed	17,770 (87.3%)	21,762 (84.5%)
Time for 5 sits and stands (s)	11.2 ± 8.0	12.2 ± 8.4
Time for CST-5 > 15 s		
Yes	2042 (11.5%)	3409 (15.7%)
No	15,728 (88.5%)	18,353 (84.3%)

*ISCED-97* International Standard Classification of Education 1997, *BMI* Body mass index, *CST-5* Five repetition chair-stand test, Mean ± standard deviation, n (%)

^a^Primary education

^b^Lower secondary education

^c^(Upper) secondary education

^d^Post-secondary non tertiary education

^e^First stage of tertiary education

^f^Second stage of tertiary education

In the crude model, increased risks of diabetes were found for persons who considered the CST-5 as not safe, or whose times for five rises were in the highest or second highest quartiles (relative risks 2.18 (95% CI: 1.95–2.43), 1.71 (1.54–1.91), 1.44 (95% CI: 1.29 –1.61)) (Table 2). These associations were attenuated in the fully adjusted regression model (relative risks 1.32 (95% CI: 1.17–1.48), 1.23 (1.10–1.37), 1.19 (1.06–1.33)).

**Table 2:** Relative risks with 95% confidence intervals for the associations between the results of the chair-stand test and incidence of type 2 diabetes: the Survey of Health, Ageing and Retirement in Europe (SHARE)

Chair-stand test	N	n (%)^**c**^	Person years	Incidence rates per 1000 person years	Relative risks (95% CI)		
					Model 1	Model 2	Model 3
Not safe^a^	6587	725 (11.0%)	32,480	22.3	2.18 (1.95–2.43)	2.11 (1.88–2.36)	1.32 (1.17–1.48)
Q1 (≥ 12.01 seconds in men, ≥ 13.5 seconds in women)^b^	9893	857 (8.7%)	51,310	16.7	1.71 (1.54–1.91)	1.65 (1.48–1.85)	1.23 (1.10–1.37)
Q2 (≥ 10.0, < 12.01 seconds in men; ≥ 10.54, < 13.5 seconds in women)^b^	10,161	739 (7.3%)	51,953	14.2	1.44 (1.29–1.61)	1.40 (1.25–1.57)	1.19 (1.06–1.33)
Q3 (≥ 8.0, < 10.0 seconds in men; ≥ 8.24, < 10.54 seconds in women)^b^	9806	595 (6.1%)	53,648	11.1	1.20 (1.07–1.35)	1.19 (1.06–1.33)	1.08 (0.96–1.21)
Q4 (reference) (< 8.0 seconds in men, < 8.24 seconds in women)^b^	9672	489 (5.1%)	54,256	9.0	1	1	1

Model 1: crude

Model 2: adjusted for age and sex

Model 3: adjusted for age, sex, BMI, physical activity, number of chronic diseases, education, country

*CI* Confidence interval, *Q1, Q2, Q3, Q4* Time needed for the CST-5 in seconds in the highest (Q1), second highest (Q2), second lowest (Q3), or lowest quartile (Q4)

^a^Participants considered the test as not safe and did not perform the test

^b^Time in seconds refers to the time needed for 5 sits and stands in the chair-stand test

^c^Cases of incident diabetes

In the fully adjusted models, the risk of diabetes in obese persons did not depend on whether they had low muscle strength or not (Tables 3 and 4). Regardless of how they performed in the CST-5, obese persons had a risk of type 2 diabetes which was 2.8 or 2.9 times higher than the risk of non-obese persons with short times in the CST-5 (Table 3). In obese persons with times for 5 sits and stands > 15 seconds, the risk of diabetes was 2.56 (95% CI: 2.22–2.95) times higher than in non-obese persons with times ≤15 seconds. The corresponding relative risk in obese persons with times ≤15 seconds was 2.45 (2.25–2.67) (Table 4).

**Table 3:** Relative risks with 95% confidence intervals for the associations between the results of the chair-stand test (four categories), presence of obesity and incidence of type 2 diabetes: the Survey of Health, Ageing and Retirement in Europe (SHARE)

BMI	Time for CST-5	N	n (%)^**c**^	Relative risks (95% CI)		
				Model 1	Model 2	Model 3
≥ 30 kg/m^2^	Not safe^a^	1657	288 (17.4%)	4.41 (3.80–5.12)	4.27 (3.67– 4.97)	2.85 (2.43–3.35)
≥ 30 kg/m^2^	Q1 (≥ 12.01 seconds in men, ≥ 13.5 seconds in women)^b^	2116	321 (15.2%)	3.85 (3.33–4.45)	3.72 (3.21–4.31)	2.86 (2.46–3.32)
≥ 30 kg/m^2^	Q2-3 (≥ 8.0, < 12.01 seconds in men; ≥ 8.24, < 13.5 seconds in women)^b^	3508	484 (13.8%)	3.50 (3.06–4.00)	3.42 (2.99–3.91)	2.94 (2.57–3.37)
≥ 30 kg/m^2^	Q4 (< 8.0 seconds in men, < 8.24 seconds in women)^b^	1250	157 (12.6%)	3.19 (2.66–3.82)	3.15 (2.63–3.77)	2.76 (2.30–3.31)
< 30 kg/m^2^	Not safe^a^	4930	437 (8.9%)	2.25 (1.96–2.58)	2.12 (1.84–2.45)	1.53 (1.32–1.77)
< 30 kg/m^2^	Q1 (≥ 12.01 seconds in men, ≥ 13.5 seconds in women)^b^	7777	536 (6.9%)	1.75 (1.53–2.00)	1.66 (1.45–1.90)	1.37 (1.20–1.58)
< 30 kg/m^2^	Q2-3 (≥ 8.0, < 12.01 seconds in men; ≥ 8.24, < 13.5 seconds in women)^b^	16,459	850 (5.2%)	1.31 (1.16–1.48)	1.27 (1.13–1.44)	1.19 (1.05–1.35)
< 30 kg/m^2^	Q4 (< 8.0 seconds in men, < 8.24 seconds in women)^b^	8422	332 (3.9%)	1	1	1

Model 1: crude

Model 2: adjusted for age and sex

Model 3: adjusted for age, sex, physical activity, number of chronic diseases, education, country

*CST-5* Five repetition chair-stand test, *Q1, Q2, Q3, Q4* Time needed for the CST-5 in seconds in the highest (Q1), second highest (Q2), second lowest (Q3), or lowest quartile (Q4), *CI* Confidence interval

^a^Participants considered the test as not safe and did not perform the test

^b^Time in seconds refers to the time needed for 5 stands in the chair-stand test

^c^Number of persons (%) with incident type 2 diabetes

**Table 4:** Relative risks with 95% confidence intervals for the associations between the results of the chair-stand test (two categories), presence of obesity and incidence of type 2 diabetes: the Survey of Health, Ageing and Retirement in Europe (SHARE)

BMI	Time for CST-5	N	n (%)^**a**^	Relative risks (95% CI)		
				Model 1	Model 2	Model 3
≥ 30 kg/m^2^	> 15 s	1228	198 (16.1%)	3.27 (2.85–3.75)	3.28 (2.86–3.77)	2.56 (2.22–2.95)
≥ 30 kg/m^2^	≤ 15 s	5646	764 (13.5%)	2.75 (2.53–2.99)	2.75 (2.53–2.99)	2.45 (2.25–2.67)
< 30 kg/m^2^	> 15 s	4223	317 (7.5%)	1.52 (1.35–1.71)	1.49 (1.33–1.68)	1.27 (1.12–1.43)
< 30 kg/m^2^	≤ 15 s	28,435	1401 (4.9%)	1	1	1

Model 1: crude

Model 2: adjusted for age and sex

Model 3: adjusted for age, sex, physical activity, number of chronic diseases, education, country

*CST-5* Five repetition chair-stand test

^a^Number of persons (%) with incident type 2 diabetes

Addition of the results of the CST-5 to a logistic regression model including age, sex, BMI, vigorous physical activity, number of chronic diseases, and ISCED led only to a very slight increase of the AROC. For the original model, AROC was 0.6888, which increased to 0.6916 after adding the time for performing the CST-5 (the improvement of AROC was 0.00274 (95% CI: 0.00117–0.00431)).

Cramer’s V for the association between the five-level categorical variables of the CST-5 and the HGST was 0.25, the Pearson coefficient between handgrip strength and time for five stands in the CST-5 was − 0.143.

## Discussion

To our knowledge, this is the first study to show that poor results in the CST-5 are associated with an increased risk of type 2 diabetes in the older population. However, we could not show that persons who are obese and show a poor result in the CST-5 have a higher risk of diabetes than persons with obesity alone. Moreover, CST-5 only leads to a negligible improvement of diabetes prediction when added to a regression model including strong risk factors of diabetes. Finally, our study shows that results of the CST-5 are only poorly to moderately correlated with results of the HGST.

### Comparison with earlier studies

A recent cross-sectional study on the one leg stand-up test in Japanese males also showed that poor lower body muscle strength is a risk factor for diabetes [30]. Participants unable to stand on right and left legs had a larger odds of diabetes than participants succeeding to stand on both right and left leg (Odds Ratio = 1.37 (95% CI: 1.04– 1.81)).

Sarcopenic obesity, i.e. the combination of sarcopenia and obesity, has been shown to be associated with higher risks of cardiovascular diseases and mortality than sarcopenia or obesity alone [31]. Sarcopenia is defined by the age-related loss of muscle mass and muscle strength, but as muscle strength declines faster in aging persons than muscle mass, the combination of obesity and low muscle strength alone (called “dynapenic obesity”) was considered as a risk factor for type 2 diabetes [12, 32]. In the English Longitudinal Study of Ageing, obese persons with handgrip strength in the high, intermediate and low tertile were compared to non-obese persons with handgrip strength in the high tertile (HR = 4.93 (95% CI: 2.85–8.53) (obese, low handgrip strength), HR = 4.85 (2.90–8.11) (obese, intermediate handgrip strength), and HR = 3.25 (1.89–5.60) (obese, high handgrip strength, respectively)) [12]. Thus, the well-known effect of obesity on diabetes risk was modified by handgrip strength, and obese persons with low or intermediate handgrip strength had a higher diabetes risk than obese persons with high muscle strength. This effect modification was not seen in the present study. Strictly speaking, there was even a small antagonistic effect in the present study because the diabetes risk in obese persons with poor results in the CST-5 was even slightly smaller than the sum of the risks of those who were only obese or only had poor CST-5 results. In a cross-sectional study from South Korea, type 2 diabetes was strongly associated with sarcopenic obesity (OR = 2.16 (1.08–3.27)), but not with sarcopenia alone (OR = 1.24 (0.86–2.15)) (reference: non-sarcopenia) [33]. However, in that study, persons with sarcopenic obesity were not compared to obese persons without sarcopenia, and, thus, it was unclear whether sarcopenia modified the diabetes risk of persons with obesity.

The present study showed that poor lower body muscle strength is not suitable as an independent variable in prediction models for type-2 diabetes. This in line with an earlier study showing that adding handgrip strength to an existing model for diabetes prediction does not lead to a relevant improvement of the predictive performance of the model [18]. This is the more remarkable because the AROCs of the models to which muscle strength was added were rather low (< 0.70). In the latter study, Harrell’s C was improved by less than 0.001, in the present study, the AROC was improved by 0.0027. Obviously, muscle strength is not a suitable variable for diabetes prediction models.

Yeung et al. compared handgrip strength with knee extension strength, and found low Pearson correlation coefficients for healthy persons in the range of 0.35 to 0.45 [17]. Only for geriatric patients, Pearson correlations were moderate (0.44 for men, 0.57 for women). The authors drew the conclusion that handgrip strength is not a measure of whole body muscle strength. This is in accordance with findings of the present study which showed that results of the HGST and results of the CST-5 are only weakly correlated.

Several mechanisms for the association between lower limb muscle strength and diabetes risk were suggested. Reduced quadriceps muscle strength may be associated with increased homeostasis model assessment of insulin resistance (HOMA-IR) [34]. Lower limb muscle strength may attenuate walking function, and thus lead to lower physical activity which in turn leads to higher diabetes risk. Moreover, skeletal muscles are an important site for the disposal of glucose, and, thus, weaker muscles which are smaller take up less glucose [35].

### Clinical relevance of the results

The adjusted relative risks indicate an, albeit small, effect of poor CST-5 results on diabetes (cf. Table 2, model 3). However, for clinical purposes, it is often equally important to identify persons with a high risk of disease, regardless of whether the underlying relation is causal, and in this case, crude associations are useful, too [36]. Given the strong crude and age-sex associations between CST-5 and diabetes, persons with poor test results have an increased diabetes risk, and, as known from earlier studies, also have increased risks for mortality and other diseases [9–11]. Nevertheless, the BMI is better at identifying persons at risk of diabetes than the CST-5: the CST-5 does not improve diabetes prediction models which include conventional risk factors like the BMI, and, moreover, the high diabetes risk of obese persons is not modified by the CST-5.

### Limitations and strengths

Our study has several limitations. First, this is an observational study so that we cannot show that the association between CST-5 and diabetes is causal. In particular, there may be residual confounding in variables of the adjustment set. Therefore, we cannot state that improvements in the CST-5 delay or prevent the onset of type 2 diabetes. Second, the assessment of chronic diseases was based on self-report. In particular, assessment of diabetes relied on self-reports (of a doctor’s diagnosis, or of the intake of diabetes drugs) rather than on measurements of HbA1c or glucose concentrations. Third, BMI was calculated on self-reported data of height and weight which may lead to some misclassification of obesity defined as BMI ≥ 30 kg/m^2^. A strength of the study is the large study size which allows for a fairly precise estimation of the effect measures. Moreover, the study sample is diverse including representative subsamples from 20 nations, and the methods of data collection are standardized in all panels and for all participating nations. Furthermore, we used different adjustment sets (crude, age-sex specific, full adjustment) to fit regression models. Finally, this is a longitudinal study contrary to many cross-sectional studies on the same topic.

## Conclusion

Poor results in the CST-5 are associated with an increased risk of diabetes. Among obese persons, the risk of diabetes is not modified by results of the CST-5. Results of the CST-5 are not suitable to improve prediction tools for type-2 diabetes.

## Data Availability

The datasets analyzed during the current study are publicly available to the entire research community free of charge: http://www.share-project.org/data-access.html.

## Abbreviations

AROC: Area under the receiver operating characteristic curve
BMI: Body mass index
CI: Confidence interval
CST: Chair-stand test
CST-5: Five-repetition chair-stand test
CST-30: 30 second chair-stand test
EWGSOP2: European Working Group on Sarcopenia in Older People
HGST: Handgrip strength test
HR: Hazard ratio
ISCED: International Standard Classification of Education
OR: Odds ratio
SHARE: Survey of Health, Ageing and Retirement in Europe
